# Supplementary material for: Assessment Tools of Biopsychosocial Frailty Dimensions in Community-Dwelling Older Adults: A Narrative Review
Source: Int J Environ Res Public Health. 2022 Nov 30;19(23):16050. doi: 10.3390/ijerph192316050 (PMC9739796; doi:10.3390/ijerph192316050)
Supplement: Supplementary file 1 [file ijerph-19-16050-s001.zip › ijerph-2009414-supplementary.pdf]

	START/STOPP	STOPPFrail	Beers criteria	Norwegian General Practice criteria (NORGEP)	(EU) (7)-PIM list	PRISCUS list	FORTA (Fit fOR The Aged) list	MAI (Medication Appropriateness Index)	STRIP method (Systematic Tool to Reduce Inappropriate	GP-GP (good palliative-geriatric practice)	IMAP (individualised medication assessment and	Zhan Criteria	Drug Regimen Unassisted Grading Scale (DRUGS)	Medication Management Ability Assessment (MMAA)	Self-Efficacy for Appropriate Medication Use Scale (SEAMS)
Sadowski C.A., et al. 2021 [142]	X														
Saeed D., et al. 2021 [143]	X	X													
Amorim W.W., et al. 2021 [144]	X		X	X	X	X									
Loste C., et al. 2021 [145]	X		X												
Pazan F., et al. 2021 [146]	X		X				X								
De Oliveira L.M., et al. 2021 [147]			X												
Williams S., et al. 2019 [148]	X		X				X								
Muth C., et al. 2019 [149]	X		X		X		X	X	X						
Kok R.M., et al. 2017 [150]	X														
Huiskes V.J., et al. 2017 [151]	X							X							
Ulley J., et al. 2019 [152]	X		X								X				
Storms H., et al. 2017 [153]	X		X			X		X							
Mohamed M.R., et al. 2020 [154]	X							X				X			
Cross A.J., et al. 2020 [155]													X	X	X
Palmer K., et al. 2019 [156]	X						X								
Muhlack D.C., et al. 2017 [157]			X												
Hasan Ibrahim A.S., et al. 2021 [158]	X									X					

Kua C.H., et al. 2019 [159]	X		X												
Ibrahim K., et al. 2021 [160]	X	X	X												
Chang C.T., et al. 2021 [161]	X		X												
	**17**	**2**	**12**	**1**	**2**	**2**	**4**	**4**	**1**	**1**	**1**	**1**	**1**	**1**	**1**

Table S1: assessment tools of prescription appropriateness and adherence in older adults' population

	PREDIMED	10-point Mediterranean-diet scale	Mini Nutritional Assessment (MNA)	Mini Nutrition Assessment Short Form (MNA-SF)	Canadian Nutrition Screening Tool (CNST)	Council on Nutrition Appetite Questionnaire (CNAQ)	DETERMINE Checklist	Malnutrition Screening Tool (MST)	Malnutrition Universal Screening Tool (MUST)	SCALES (Sadness, Cholesterol, Albumin, Loss of weight, Eating, Shopping) questionnaire	Seniors in the Community Risk Evaluation for Eating and Nutrition questionnaire (SCREEN I) or (SCREEN II)	Simplified Nutritional Appetite Questionnaire (SNAQ)	Nutritional Form for the Elderly (NUFFE)	Chapman Screening tool	Short Nutritional Assessment Questionnaire (SNAQ65+)
Suárez M et al. 2017 [162]	x	x													
Laur CV et al. 2017 [163]			x		x										
Dwyer JT et al. 2020 [164]			x	x		x	x	x	x	x	x	x			
Marshall S. 2016 [165]				x				x	x				x	x	x
Zupo R et al. 2020 [166]				x											
Barchitta M et al. 2020 [167]	x														
Oktaviana J et al. 2020 [168]			x												

Robertson DA et al. 2013 [169]	x														
Cox et al. 2020 [170]												x			
Jadczak AD & Visvanathan R. 2019 [171]															
Fernández-Pombo A et al. 2021 [172]			x						x			x			
Ruiz M & Kamerman LA, 2010 [173]			x				x		x						
Kuzuya M, 2021 [174]			x												
	3	1	6	3	1	1	2	2	4	1	1	3	1	1	1

Table S2: assessment tools of nutritional habits

	6-minute walking test (6MWT)	SF-36 (physical component scale)	5-meter walking test (5MWT)	Short Physical Performance Battery (SPPB)	Physical Activity Scale for the Elderly (PASE)	Community Healthy Activities Model Program for Seniors CHAMPS	Zutphen Physical Activity Questionnaire	Physical Activity and Sedentary Behavior Questionnaire (PASB-Q)	EPIC Physical Activity Questionnaire (EPAQ2)	International Physical Activity Questionnaire (IPAQ)	Stanford Brief Activity Survey (SBAS)	Women’s Health Initiative Physical Activity Questionnaire (WHI-PAQ)	GPPAQ General Practice Physical Activity Questionnaire	Longitudinal Aging Study Amsterdam Physical Activity Questionnaire (LAPAQ)	PASIPD, Physical Activity Scale for Individuals with Physical Disabilities	Duke’s activity status index (DASI)	Incremental Shuttle Walk Test (ISWT)	WOMAC physical function sub-scale	5CST, five-chair stand test	Timed-Up & Go Test	Four-Square Step test.	Tinetti Performance Oriented Mobility Assessment (POMA)	Alternate-step test (ATS)	Mini Motor Test (MMT)	Elderly Mobility Scale
Giannitsi S. et al. 2019 [175]	x																								
Vadalà G. et al. 2020 [176]		x																							
Tamuleviciute-Prasciene E et al. 2018 [177]			x																						
Lynch EA et al. 2018 [178]	x																								

McGettigan M et al. 2020 [179]		x		x																					
Gordt K et al. 2018 [180]		x		x	x																				
Jian-Yu E et al. 2020 [181]					x	x																			
Schwenk M et al. 2014 [182]				x																					
Albrecht M et al. 2018 [183]							x																		
Sattler MC et al. 2020 [184]					x			x	x	x	x	x	x	x											
Karpman C & Benzo R., 2014 [185]	x																								
Vialleron T et al. 2020 [186]	x																								
Aquilani R et al. 2014 [187]	x																								
Berner K et al. 2017 [188]				x																					
You Y et al. 2021 [189]	x			x																					
Donini LM et al. 2016 [190]	x																								

Cheng DK et al. 2021 [191]	x			x											x										
Argillander TE et al. 2021 [192]	x			x																					
Qiu S et al. 2015 [193]										x															
Daami M et al. 2017 [194]	x																								
Keating CJ et al. 2021 [195]				x																					
Mehdizadeh D et al. 2021 [196]				x																					
Vavasour G et al. 2021 [197]				x																					
Pu C.Y. et al. 2021 [198]	x																								
Baritello O et al. 2021 [199]	x			x																					
Anabitarte-García F et al. 2021 [200]				x																					
Abou L et al. 2021 [201]	x			x																					
Elena P et al. 2021 [202]				x																					

Mañas A et al. 2021 [203]	x			x	x																				
Yoo J et al. 2021 [204]	x			x												x	x								
Pitsillides A et al. 2021 [205]				x														x							
van der Kruk E et al. 2021 [206]	x			x																					
Williams F.R et al. 2021 [207]	x			x																					
Bortone I et al. 2021 [208]	x			x															x	x					
Inoue T et al. 2021 [209]																				x	x				
Yang F et al. 2021 [210]				x																x					
Gielen E et al. 2021 [211]	x			x																					
Pazan F et al. 2021 [146]				x																x					
Gafner S.C et al. 2021 [212]				x																x		x			
McMillan J.M. et al. 2021 [213]																				x					
Ponciano V et al 2021 [214]																				x					

Lund C.M et al. 2020 [215]	x			x	x																				
Zheng L et al. 2020 [216]				x																x					
Guo Z et al. 2020 [217]																				x					
Byra J & Czernicki K. 2020 [218]	x																								
Zampogna B et al. 2020 [219]	x																	x							
Oktaviana J et al 2020 [168]				x																					
Wang D.X.M et al 2019 [220]	x			x																x		x			
Chen M.Y et al. 2019 [221]	x																								
Pei G et al 2019 [222]	x																								
Bet P et al 2019 [223]	x			x																x		x	x	x	
Granic A. et al. 2019 [224]				x																					
Facal D et al. 2019 [225]				x																x					
Mol A et al. 2018 [226]																				x					

Wright J. et al. 2018 [227]																				x					
Liao C et al. 2018 [228]	x			x	x																	x			
Doma K et al. 2018 [229]	x																			x					
Lopez P. et al. 2018 [230]				x																x					
Dewansingh P et al. 2018 [231]	x			x																x					
Luo D et al. 2017 [232]				x																x					
Apóstolo J et al 2017 [233]																				x					
Wouters H et al. 2017 [234]				x																x					
van Deudekom F.J et al. 2017 [235]																				x					
Bahureksa L et al. 2017 [236]																				x					
McDonald V.S et al. 2016 [237]				x																x					
Bullo V et al. 2015 [238]																				x	x				
Furukawa & H.,	x																								

Tanemoto K. 2015 [239]																									
McNeely M.E et al 2015 [240]																				x		x			
Hill K.D. et al 2015 [241]					x															x					
Pamoukdjian F et al. 2015 [242]				x																x					
Stehr M.D et al. 2012 [243]		x			x	x																			
Chou C.-H et al. 2012 [244]		x																		x					x
Lee P et al. 2012 [245]				x		x																			
Den Ouden M.E.M et al. 2011 [246]																						x			
	33	5	1	40	8	3	1	1	1	2	1	1	1	1	1	1	1	2	1	30	2	6	1	1	1

Table S3: assessment tools of physical activity

	Falls Efficacy Scale (FES)	Modified Falls Efficacy Scale (MFES)	Activities-specific Balance Confidence scale (ABC)	Survey of Activities and Fear of Falling in the Elderly (SAFE)	Fall risk assessment tool (FRAT)	Mobility Efficacy Scale (MES)	Short Falls Efficacy Scale-International (FES-I)	University of Illinois at Chicago Fear of Falling Measure	Fall Risk Index	Fracture Risk Assessment Tool (FRAX)	Tinetti Performance-Oriented Mobility Assessment (POMA)	Berg Balance Scale	Morse Fall Scale	Downton Fall Risk Index	St. Thomas Risk Assessment Tool in Falling in Elderly Inpatients	Conley Scale	Falls Risk for Older Persons-Community Setting Screening Tool (FROP Com)	Johns Hopkins Fall Risk Assessment Tool (JHFRAT)	Fullerton Advanced Balance (FAB) Scale	Hendrich II Fall Risk Model	Austin Health Falls Risk Screening Tool (AHFRST)	Elderly Falls Screening Tool	Home-Screen Scale	Safety House Checklist	Falls Risk for Hospitalized Older People (FRHOP)	Falls Risk Awareness Questionnaire (FRAQ)
Choi SD et al. 2017 [247]	x																									
Kendrick D et al. 2014 [248]	x	x	x	x																						
Papalia GF et al. 2020 [249]	x		x																							
De Coninck L et al. 2017 [250]	x		x																							

Nunan S et al. 2018 [251]					x																					
Kumar A et al. 2016 [252]	x		x	x		x																				
E JY et al. 2020 [181]							x	x																		
Bullo V et al. 2015 [238]									x																	
Liau SJ et al. 2021 [253]																										
Zhang J et al. 2020 [254]										X																
Strini V et al. 2021 [255]			x				x				x	x	X	x	X	x	x	x	x	x	x					
Zheng L et al. 2020 [216]			x				x																			
Zengin A et al. 2018 [256]																						x				

Lee J et al. 2013 [257]													X		x											
Romli MH et al. 2017 [258]																							x	x		
Williams-Roberts H et al. 2020 [259]	x				x						x	x	x		x		x	x		x					x	
Omaña H et al. 2021 [260]											x	x														
Wiseman T & Betihavas V, 2019 [261]	x																									
Vilaca T et al. 2020 [262]										X																
Howcroft J et al. 2013 [263]											x				x											
Ciorba A et al. 2017 [264]			x																							

Lenouvel E et al. 2021 [265]							x																			
Anthony K et al. 2013 [266]	x																									
Aranda-Gallardo M et al 2013 [267]											x		x	x	x	x				x						
Reeve E et al. 2020 [268]							x																			
Wang J et al. 2010 [269]	x	x	x	x																						
Bahat G et al. 2021 [270]										X																
Senderovich H et al. 2021 [271]											x															
Kruisbrink M et al. 2021 [272]	x		x	x			x																			

Lim SER et al. 2021 [273]							x																			
Sattar S et al 2020 [274]																				x						
Kruisbrink M et al. 2021 [275]	x		x	x			x																			
Stockwell-Smith G et al. 2020 [276]																				x						
McMahon S et al. 2012 [277]	x	x	x	x		x																				
Martins Gaspar AG & Lapão LV, 2021 [278]	x											x														
Hill A et al. 2019 [279]	x				x										x					x						x
Lopez P et al. 2018 [230]	x		x																							

Marques-Vieira CMA et al. 2016 [280]							x																		
Visschedijk J et al. 2010 [281]	x		x									x													
	16	3	13	6	3	2	9	1	1	1	6	5	4	2	6	2	2	2	1	6	1	1	1	1	1	1

Table S4: fall and the risk of falling assessment tools

	Mini Mental State Examination (MMSE)	Short portable mental status questionnaire (SPMSQ or Pfeiffer)	Abbreviated Mental Test (AMT)	Montreal Cognitive Assessment (MoCA)	Clinical Dementia Rating scale (CDR)	Controlled Oral Word Association Test (COWAT)	Trail Making Test A & B (TMT)	Rey Auditory Verbal Learning Test [illegible]	Clock drawing test (CDT)	Isaac Set Test (IST)	Cognitive Abilities Screening Instrument (CASI)	Brief Cognitive Screening Battery (BCSB)	Modified Mini Mental State Examination (3MS)	Clifton Assessment Procedures for the Elderly (CAPE)	Telephone Interview for Cognitive status (TICS)	Cognitive screening instrument for dementia (CSID)	6-item cognitive impairment test (6-CIT)	Mini-cog
Caçador C et al., 2021 [282]	X	X																
van Aalst FM et al., 2020 [283]			X	X														
Facal D et al., 2019 [225]	X			X	X	X	X	X	X									
Borges MK et al., 2019 [284]	X			X	X					X	X							

Panza F et al., 2018 [285]	X			X	X													
van Deudekom FJ et al., 2017 [235]	X																	
Panza F et al., 2014 [286]	X	X								X		X	X					
Robertson DA et al., 2013 [169]	X	X								X		X	X					
Gracie TJ et al., 2021 [287]	X			X														
Zheng L et al., 2020 [288]	X			X	X					X								
Vella Azzopardi R et al., 2018 [289]	X	X												X	X	X	X	X
van Deudekom FJ et al., 2017 [235]	X																	
	11	4	1	6	4	1	1	1	1	4	1	2	2	1	1	1	1	1

Table S5: assessment tools for cognitive function

	UCLA Loneliness scale	De Jong Gierveld scale	Three Item Loneliness Scale	Social Frailty Index (SFI)	Questionnaire to define Social Frailty Status (QSFS)	Chicago Social Activity Scale	Life Space Index (LSI)	Steptoe Social Isolation Index	11-items Duke Social Support Index
Courtin E, Knapp M. 2017 [290]	x	x							

Cohen-Mansfield J. et al. 2015 [291]	x	x							
Li J et al. 2018 [292]	x								
Smith KJ, & Victor C, 2021 [293]	x	x							
van der Aa H.P. et al. 2016 [294]	x								
McClelland H. et al. 2020 [295]	x	x							
Noone C. et al. 2020 [296]	x								
Chawla K et al. 2021 [297]	x	x	x						
Casanova G. et a. 2021 [298]	x	x							
Ellis S. et al. 2021 [299]	x	x							
Krzeczkowska A. et al. 2021 [300]	x								
Bessa B, et al. 2018 [301]				x	x				
Landeiro F, et al. 2017 [302]	x	x							x
Tong F. et al. 2021 [303]	x	x				x	x	x	
Dahlberg L. et al. 2020 [304]	x	x							
	16	11	1	1	1	1	1	1	

Table S6: loneliness assessment tools

	UCLA Loneliness Scale	OARS Multidimensional Functional Assessment of Older Adults (MFAQ)	Inventory of Socially Supportive Behaviours (ISSB)	Social support behaviors (SS-B) scale	Multidimensional Scale of Perceived Social Support	Berkman-Syme Social Network Index (SNI)	De Jong Gierveld Scale	Social Provisions Scale	Personal Resource Questionnaire (PRQ)	Lubben Social Network Scale	Duke Social Support Index (DSSI)	Medical Outcome Study Social Support Survey (MOS-SSS)	Six social support deficits	2-Way Social Support Scale	Philadelphia Geriatric Center Morale Scale (PGCMS)
Lem K et al 2021 [137]	X	X	X	X	X	X	X	X	X						
Casanova G et al 2021 [298]	X						X	X		X					
Cappelli M et al 2020 [305]	X	X				X									
Tengku Mohd TAM et al 2019 [306]		X			X				X	X	X	X	X	X	
Cohen-Mansfield J et al 2015 [291]	X	X					X								X
	4	4	1	1	2	2	3	2	2	2		1	1	1	1

Table S7: assessment tools for social support network
